# Short-term surgical outcomes for patients with non-functional pancreatic neuroendocrine tumors are unaffected by chronological age

**DOI:** 10.1016/j.sopen.2026.03.002

**Published:** 2026-03-06

**Authors:** Ughur Aghamaliyev, Athanasios Zamparas, Gwendolin Seidel, Yannick Meyer, Gulnar Jafarova, Dominik Koch, Felix O. Hofmann, Dionysios Koliogiannis, Matthias Ilmer, Javad Karimbayli, Jens Werner, Bernhard W. Renz

**Affiliations:** a Department of General, Visceral and Transplantation Surgery, LMU University Hospital, LMU Munich, Germany; b German Cancer Consortium, partner site Munich, Munich, Germany; c Department of Surgery, Division of Hepatobiliary and Pancreatic Surgery, Mayo Clinic, Rochester, MN, USA; d Thumbay Research Institute for Precision Medicine, Gulf Medical University, Ajman, 4184, United Arab Emirates

**Keywords:** POPF, PPAP, ideal outcome, IO, pNET, NET, neuroendocrine tumor

## Abstract

**Background:**

Non-functional pancreatic neuroendocrine tumors (NF-pNETs) are increasingly diagnosed, particularly in older adults, largely due to widespread cross-sectional imaging. Patients with NF-pNETs typically have a soft pancreas and a small main pancreatic duct, which puts them at high risk for postoperative pancreatic fistula B/C, significantly impacting postoperative morbidity and mortality. Given the rising incidence of NF-pNETs and ongoing uncertainty about optimal treatment in older individuals, this study evaluates postoperative outcomes and histopathological differences between older and younger patient cohorts.

**Material and methods:**

In this study, a total of 171 patients that underwent any type of pancreatic resection for NF-pNETs between 2003 and 2023 at our institution, were investigated retrospectively upon demographic data, postoperative complications, duration of hospital stay and tumor histology. Patients were stratified into younger (< 70 years) and older (> 70 years) groups. Postoperative short-term outcomes were compared between both groups.

**Results:**

A total of 171 patients underwent pancreatic resection for NF-pNETs, including 129 younger (<70 years) and 42 older (≥70 years) patients. Older patients exhibited a markedly higher prevalence of cardiac (62% vs. 30%) and pulmonary conditions (15% vs. 5%) compared to their younger counterparts. Surgical approaches and tumor locations were similar. Postoperative outcomes, including POPF, PPH, DGE, LOS, and 30-day mortality, did not significantly differ between groups. Ideal outcome (IO) was achieved in 45.5% of younger and 37.2% of older patients (p = NS). Oncological features such as tumor stage, lymph node involvement, grading, and Ki-67 index were comparable.

**Conclusion:**

Surgical resection for NF-pNETs appears to be a safe option in older patients (≥70 years), as chronological age by itself does not significantly impact postoperative outcomes.

## Introduction

Pancreatic neuroendocrine tumors (pNETs) can be categorized into functional pNETs, such as insulinomas, gastrinomas, and VIPomas, and non-functional pNETs (NF-pNETs) [1]. Notably, approximately 60–90% of pNETs are non-functional, which distinguishes them from functional pNETs that secrete hormones [2]. A significant concern is that 60% of NF-pNETs present with systemic metastasis at initial diagnosis, but they tend to have a better prognosis than many other gastrointestinal cancers, with 5-year survival rates ranging from 27% to 33% in metastatic disease [3], [4]. Studies have shown that aggressive management, including surgical intervention, improves outcomes even in patients with metastatic disease. For instance, a SEER database study found that surgical resection of the primary tumor in patients with metastasis has been shown to extend overall survival, improving the median from one year to five years [3]. Surgical resection is an option for fewer than half of patients with metastatic NF-pNETs, and even among those operated on, recurrence rates remain high [3]. There is growing support for the survival benefit of surgical debulking in certain cases; however, conclusive high-level evidence is still limited.

In recent years, incidence of NF-pNETs has been rising, largely attributed to an aging population and the increased use of cross-sectional imaging techniques [5]. This rise is particularly pronounced for NF-pNETs measuring less than 2 cm, with the incidence of pNETs ≤2 cm in the United States having increased by seven-fold over the past 22 years [6]. This enormous increase raised questions and discussion about the management of these small NF-pNETs [7]. Current guidelines consistently advocate surgical removal for functional and large pNETs, yet the management of small, non-functional tumors remains uncertain and subject to ongoing discussion [8]. On one side, it has been shown that, so called incidentalomas or NF-pNETs ≤2 cm are mostly benign lesions with low malignant transformation potential and thus might be good candidates for surveillance [7], [9], [10], [11].

On the other hand, many studies have demonstrated a clear survival benefit for operated patients compared to those under active surveillance [12], [13], [14]. Indeed, patients with NF-pNETs typically have a soft pancreas and a small main pancreatic duct, which puts them at high risk for postoperative pancreatic fistula B/C (POPF B/C), significantly impacting postoperative morbidity and mortality [15]. Hence, surgical indications should be critically assessed, especially in older patient populations. However, data on postoperative outcomes in patients older than 70 years is lacking. Therefore, the primary aim of this study was to compare postoperative complication rates in patients with NF-pNET aged over 70 years versus younger cohorts. The secondary aim was to analyze histopathological differences between the two groups.

## Material and methods

### Study population and inclusion/exclusion criteria

This single-center retrospective cohort study included patients who underwent any type of pancreatic resection for NF-pNETs at our institution between 01/2003 and 12/2023. The study was conducted with approval of the local ethics committee (reference number: 24-0014). The analysis included patients who were 18 years or older and had undergone elective pancreatoduodenectomy (PD), distal pancreatectomy (DP) or enucleation. Exclusion criteria were functional pNETs, other types of pancreatic malignancies, individuals who had undergone previous pancreatic surgery, and patients with missing data. Patients 70 years of age and older were compared to younger ones.

### Data collection

Preoperative variables collected included demographic information, body mass index (BMI), ASA classification (American Society of Anesthesiologists), history of alcohol abuse, smoking status, presence of cardiovascular and pulmonary comorbidities, preexisting diabetes and prior administration of neoadjuvant therapy. Cardiac comorbidity included, arterial hypertension, chronic heart failure, atrial fibrillation, coronary artery disease, history of acute myocardial infarction, aortic valve insufficiency, mitral valve insufficiency, aortic valve stenosis, and history of aorto-coronary bypass surgery while pulmonary comorbidity included asthma, chronic obstructive pulmonary disease pulmonary hypertension, lung emphysema, and chronic bronchitis. Intraoperative variables encompassed the type of surgery performed, operative duration, estimated blood loss, intraoperative blood transfusion requirements and whether splenectomy, multivisceral resection, or vascular resection were performed. Postoperative outcomes included histopathological findings, length of hospital stay, and any postoperative complications, with a focus on clinically significant issues such as POPF, delayed gastric emptying (DGE), and post-pancreatectomy hemorrhage (PPH). 30-day mortality rates were also assessed. The Ideal Outcome was defined as the absence of major complications (Clavien-Dindo grade ≥ 3a), POPF B or C according to ISGPS, prolonged hospital stays beyond the 75th percentile, hospital readmission, reoperation, and postoperative mortality [16].

### Summary of standard operative protocol

For patients diagnosed with tumors of the pancreatic head, an open PD was performed, while DP was undertaken for tumors located within the body to tail. The ISGPS consensus statement was utilized to determine the standard lymphadenectomy protocol for both PD and DP [17]. Enucleation was performed in selected patients according the ENETS 2017 guidelines [15]. To ensure complete resection, vascular resections and reconstructions were performed in cases of malignancies infiltrating the portal vein (PV), superior mesenteric vein (SMV) or celiac axis. All patients were administered a single intravenous prophylactic dose of antibiotics prior to incision. The postoperative use of somatostatin analogues as well as the duration of the use was determined by operating surgeon.

The reconstruction following PD was performed using pancreatojejunostomy (PJ) in accordance with standard oncological procedures. This included either the classical partial pancreaticoduodenectomy, as described by Kausch-Whipple, or the pylorus-preserving pancreaticoduodenectomy (PPPD) according to Traverso-Longmire. The intestinal reconstruction involved retro-colic placement of the first jejunal loop to facilitate an end-to-side pancreatico-enteric anastomosis. Subsequently, the bilioenteric end-to-side anastomosis was created 7–10 cm distal to the pancreaticojejunostomy. This was followed by an ante-colic gastrojejunostomy, which included a Braun anastomosis, or a pylorojejunostomy in cases where the pylorus was preserved. In the majority of patients, two drains were placed as standard practice, positioned ventrally and dorsally to the PJ. DP was conducted using either an open or minimal-invasive approach and utilizing either a stapling device or a scalpel dependent on the thickness of pancreatic parenchyma. In cases where scalpel transection was used, the pancreatic duct was identified and subsequently closed with monofilament absorbable sutures. Abdominal drains were inserted at the end of the procedure and levels of drain lipase and amylase were measured in accordance with clinic standards. The drain fluid enzyme values were routinely measured on PODs 2 & 5. The drains were removed once levels were back to normal. By patients with persistent POPF B/C till discharge day, the patient would be discharged with the abdominal drains and would be subject to weekly ambulant control. POPF B/C is defined using the latest classification provided by the ISGPS [18]. Specifically, biochemical leak was identified as an elevation in drain amylase levels without any clinical impact. Patients exhibiting prolonged drainage for over 3 weeks due to heightened amylase activity were categorized as POPF grade B. The classification of POPF grade B was also conferred when interventions such as percutaneous, endoscopic, or angiographic procedures became necessary. In cases in which a grade B POPF resulted in organ failure or clinical instability to the extent that reoperation is imperative, the POPF B is upgraded to grade C. Further, postoperative management protocol including naso-gastric tube (NGT) removal (after day 1), return of oral feeding, mobilization of peri-pancreatic drainage, and pain relief were left to the discretion of surgeons and anesthesiologists in charge of the patients. However, return of oral feeding included routinely liquid diet at POD 1or 2. Then, if well tolerated, a semi-solid diet was given at POD 2–3. No prokinetic agent was given as prophylaxis. Management of DGE was left to the discretion of surgeons.

### Statistical analysis

Categorical variables were compared using the chi-squared test (with Yates' continuity correction for 2 × 2 tables) and Fisher's exact test, and the Mann-Whitney *U* test and Student's *t*-test were used to compare continuous variables as appropriate. Univariate logistic regression analysis was used to obtain odds ratios and 95% confidence intervals (CIs) and to examine the association between perioperative clinicopathological factors and Ideal outcome. Statistical analysis and graphical illustrations were conducted using R-Software (Version 4.2.2).

## Results

### Patient demographics

A total of 171 patients who underwent pancreatic resection for NF-pNETs were included in the study, with 129 (75%) patients in the younger group (<70 years) and 42 (25%) in the older group (≥70 years). Table 1 presents a comparative analysis of patient characteristics between younger and older group. The median age was significantly higher in the older group compared to the younger group (73 vs 54, *p* < 0.001). The gender distribution was similar between the groups, with males accounting for 53% in both cohorts (*p* = 0.9). BMI did not differ significantly between groups (median 25 kg/m^2^ vs. 26 kg/m^2^, *p* = 0.6). However, older patients had significantly higher rates of cardiac diseases (62% vs. 30%, *p* < 0.001) and pulmonary diseases (15% vs. 5%, *p* = 0.03), while rates of diabetes mellitus, smoking, alcohol abuse, and neoadjuvant therapy were similar between the groups (all *p* > 0.05).

**Table 1:** Patients' characteristics.

Parameter	<70 years n = 129 (75%)	≥70 years n = 42 (25%)	*p* value
Age, [years], median (IQR)	54 (46–61)	73 (71–76)	<0.001
Male, n (%)	68 (53%)	23 (53%)	0.8
BMI, [kg/m^2^] median (IQR)	25 (22–28)	26 (22–27)	0.6
ASA ≥3, n (%)			0.8
1	1 (1%)	0 (0%)	
2	22 (24%)	8 (22%)	
3	70 (75%)	34 (78%)	
Smoker, n (%)	19 (15%)	5 (10%)	0.6
Alcohol abuse, n (%)	6 (5%)	3 (7%)	0.5
Diabetes mellitus, n (%)	19 (15%)	6 (14%)	0.9
Cardiac diseases, n (%)	39 (30%)	26 (62%)	<0.001
Pulmonary diseases, n (%)	6 (5%)	6 (15%)	0.03
Neoadjuvant therapy, n (%)	15 (10.5%)	2 (5%)	0.3

n: number; IQR: interquartile range; BMI: body-mass-index; kg: kilogram; m^2^: square meters; ASA: American Society of Anesthesiologists.

### Comparison of surgical characteristics between older and younger patients

The distribution of surgical approaches was comparable between the groups (*p* = 0.4), with PD performed in 37% of younger and 50% of older patients, DP in 53% vs. 43%, and enucleation in 10% vs. 7% (Table 2). However, vascular resection was performed exclusively in younger patients (10% vs. 0%, *p* = 0.04), while the rate of multivisceral resections was similar (19% vs. 17%, *p* = 0.7). Median operative time was similar between groups (*p* = 0.6), as were EBL (*p* = 0.2). No older patients required intraoperative blood transfusion (IBT), whereas 6% of younger patients did (*p* = 0.07). Tumor location did not significantly differ between younger and older patients (p = 0.2). In the younger cohort, tumors were predominantly located in the pancreatic tail (48%), followed by the head (41%) and body (11%). In contrast, older patients had a higher proportion of tumors in the pancreatic head (56%) and fewer in the body (6%) and tail (39%).

**Table 2:** Comparison of operative data between elderly and non-elderly patients.

Parameter	<70 years n = 129 (75%)	≥70 years n = 42 (25%)	*p* value
Tumor location			0.1
Caput	52 (40%)	24 (56%)	
Corpus	15 (12%)	2 (5%)	
Cauda	62 (48%)	16 (39%)	
Surgical approach			0.3
Pancreatoduodenectomy	47 (37%)	21 (50%)	
Distale pancreatectomy	69 (53%)	18 (43%)	
Enucleation	13 (10%)	3 (7%)	
Vascular resection, n (%)	12 (9%)	0 (0%)	0.04
Multivisceral resection, n (%)	24 (17%)	7 (17%)	0.7
Op. time [min] median (IQR)	220 (160–300)	225 (140–330)	0.6
EBL [ml], median (IQR)	400 (200–800)	375 (150–720)	0.2
IBT [ml], median (IQR)	7 (6%)	0 (0%)	0.07
LOS [days], median (IQR)	15 (11–22)	18 (13–26)	0.2

n: number; IQR: interquartile range; EBL: estimated blood loss; IBT: intraoperative blood transfusion.

### Comparison of postoperative short-term outcomes and ideal outcome

**Fig. 1:**
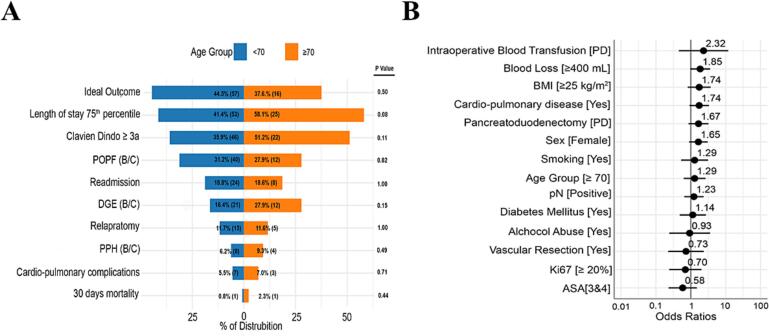
A) Comparison of ideal outcome between elderly and non-elderly patients; B) univariate analysis for ideal outcome.

The rate of POPF B/C was comparable between the two groups, occurring in 28% of older patients and 31% of younger patients (*p* = 0.1) (Fig. 1A). Similarly, the incidence of PPH B/C was not significantly different, affecting 6% of younger and 9% of older patients (*p* = 0.5). DGE B/C was numerically more frequent in older patients (28% vs. 16%), though the difference did not reach statistical significance (*p* = 0.15). The median LOS was slightly longer in older patients, but this difference was not statistically significant (18 days vs 15 days, *p* = 0.2). The 30-day mortality rate was low in both groups, with one patient in each group (0.8% vs. 2.4%, *p* = 0.4). Readmission rate was identical in both groups (18%, *p* = 0.5). Among younger patients, IO was achieved in 44.5% of cases, compared to 37.6% in older patients; however, this difference was not statistically significant. As shown in Fig. 1B, univariate analysis did not identify any significant predictors of IO (Table 3).

**Table 3:** Comparison of oncological parameters in elderly and non-elderly patients.

Parameter	<70 years n = 129 (75%)	≥70 years n = 42 (25%)	*p* value
Pathological T stage, n (%)			0.2
1	26 (21%)	13 (31%)	
2	49 (41%)	10 (24%)	
3	40 (33%)	18 (42%)	
4	6 (5%)	1 (3%)	
Tumor size [cm], median (IQR)	3 (2–4.5)	2.5 (1.4–5.2)	0.3
Pathological N stage, n (%)			0.8
0	68 (56%)	22 (60%)	
1	52 (43%)	16 (40%)	
2	1 (1%)	0 (0%)	
Resected number of LN, n (%)	14 (8–21)	11 (4–20)	0.2
Histological grade, n (%)			0.2
1	66 (52%)	24 (58%)	
2	52 (40%)	12 (29%)	
3	11 (8%)	6 (15%)	
Ki67 [%], median (IQR)	2 (1–6%)	2 (1–10%)	0.8

n: number; IQR: interquartile range; LN: lymph node.

### Comparison of oncological characteristics

The pathological tumor stage distribution showed no significant difference between the two age groups (*p* = 0.2). The proportion of patients with pT1 tumors and pT3 tumors varied between groups; however, these differences were not statistically significant. Similarly, the median tumor size did not differ significantly between older and younger patients (*p* = 0.3). Lymph node involvement was comparable between groups (*p* = 0.8), with N0 disease present in 56% of younger and 60% of older patients. The median number of resected lymph nodes was slightly lower in the older group (10.5 vs. 14), though the difference was not statistically significant (*p* = 0.2). Tumor grading showed no significant variation between groups (p = 0.2), with most patients having grade 1 or 2 tumors in both cohorts. The median Ki-67 index was identical (2% in both groups).

## Discussion

With an aging population and the growing use of cross-sectional imaging, the incidence of NF-pNETs has been steadily increasing [19], [20]. Notably older patients are more likely to present with poorly differentiated or undifferentiated tumors, which are significantly associated with worse OS [21]. Additionally, patients with pNETs often exhibit a soft pancreatic texture and a small pancreatic main duct, leading to a higher rate of postoperative complications compared to those with PDAC [22], [23]. However, we hypothesize that patients over 70 years of age may develop pancreatic fibrosis and atrophy due to aging, potentially resulting in a lower incidence of POPF B/C. To the best of our knowledge, this is the first study to compare NF-pNET patients older than 70 years with their younger counterparts.

Despite concerns regarding increased surgical risks in older patients, our study revealed that the incidence of major postoperative complications was similar between both groups. Specifically, rates of POPF B/C were similar in both cohorts, aligning with previous studies that have suggested age does not independently predict the development of POPF B/C [22]. Similarly, no significant differences were observed in PPH, reoperation rates, or 30-day mortality.

In contrast to previous meta-analyses reporting increased rates of cardiopulmonary complications in older patients [24], our study did not reveal significant differences in such events. This discrepancy may, in part, be attributed to the smaller sample size of our cohort, which may limit the statistical power to detect less frequent complications. Additionally, the absence of increased cardiopulmonary events might reflect improvements in perioperative management, careful patient selection, or the experience of high-volume surgical centers. For example, Tan et al. [25] reported a higher incidence of respiratory complications in older patients (>70 years) undergoing pancreatic resection for PDAC, although without a corresponding increase in mortality. Similarly, a study from our group found that older patients undergoing PD for PDAC had a higher burden of preoperative comorbidities, which was associated with an increased incidence of postoperative non-surgical complications [26]. Our findings suggest that with appropriate perioperative care and careful selection, older patients may not necessarily be at increased risk for cardiopulmonary complications.

Interestingly, DGE B/C was observed more frequently in older patients (27% vs. 15%), though this difference did not reach statistical significance (*p* = 0.07). This trend may be attributed to age-related physiological changes in gastrointestinal motility and a reduced capacity for postoperative adaptation. Additionally, older patients had a slightly longer median length of hospital stay (18 vs. 15 days, *p* = 0.2), which could be related to prolonged recovery times rather than increased surgical morbidity. The ideal outcome was achieved in 42.7% in whole cohort. A recently published study showed also comparable IO after pancreatic resection for pancreatic neuroendocrine tumors [27]. The rate of IO was numerically but not significantly low in older group. This is due to higher rate of DGE B/C in older patients.

From an oncological standpoint, our findings indicate no significant differences in tumor stage, lymph node involvement, tumor grade, or proliferation index (Ki-67%) between younger and older patients. While there were variations in the distribution of lower and higher pT stages between the age groups, these differences were not statistically significant. These findings contrast with previous studies that have suggested a link between increasing age and more aggressive tumor characteristics in NF-pNETs [28], [29]. For instance, Fathi et al., in a population-based analysis of 1787 patients with NF-pNETs, reported that age > 75 years was linked to increased tumor aggressiveness [28]. These discrepancies in contrast to our findings may be attributed to differences in tumor biology across populations and sample size limitations. Furthermore, improved diagnostics in detecting NF-pNETs in older patients due to increased imaging could have led to earlier diagnoses, potentially reducing the observed tumor aggressiveness in this age group. Further studies are needed to explore potential variations in tumor biology across age groups.

These findings recommend individualized decision-making based on patient comorbidities and functional status rather than age alone. Given that older patients had similar short-term surgical outcomes and tumor characteristics to younger patients, surgical resection should be considered a viable treatment option for appropriately selected older individuals.

Despite being the first investigation of its kind comparing the short-term outcomes after pancreatic resection because of NF-pNET in older (>70 years old) and younger patients, the present study is not without limitations. This study is limited by its reliance on a single-center cohort and its retrospective, non-randomized design.

In conclusion, our study suggests that older patients undergoing pancreatic resection for NF-pNETs experience comparable short term postoperative outcomes to younger patients. Age alone should not preclude surgical consideration, and careful preoperative assessment remains crucial in guiding treatment decisions.

## CRediT authorship contribution statement

**Ughur Aghamaliyev:** Writing – review & editing, Writing – original draft, Visualization, Validation, Supervision, Resources, Project administration, Methodology, Investigation, Formal analysis, Conceptualization. **Athanasios Zamparas:** Writing – original draft, Investigation, Data curation, Conceptualization. **Gwendolin Seidel:** Resources, Investigation, Formal analysis, Data curation, Conceptualization. **Yannick Meyer:** Writing – original draft, Methodology, Investigation, Formal analysis, Data curation, Conceptualization. **Gulnar Jafarova:** Writing – original draft, Resources, Methodology, Investigation, Formal analysis, Data curation, Conceptualization. **Dominik Koch:** Writing – review & editing, Writing – original draft, Investigation, Data curation, Conceptualization. **Felix O. Hofmann:** Writing – review & editing, Visualization, Methodology, Investigation, Data curation, Conceptualization. **Dionysios Koliogiannis:** Writing – review & editing, Validation, Investigation, Data curation, Conceptualization. **Matthias Ilmer:** Writing – review & editing, Methodology, Investigation, Data curation, Conceptualization. **Javad Karimbayli:** Writing – review & editing, Writing – original draft, Visualization, Validation, Methodology, Investigation, Formal analysis, Conceptualization. **Jens Werner:** Writing – review & editing, Supervision, Project administration, Methodology, Investigation, Formal analysis, Conceptualization. **Bernhard W. Renz:** Writing – review & editing, Visualization, Validation, Supervision, Resources, Project administration, Methodology, Investigation, Formal analysis, Data curation, Conceptualization.

## Ethical approval statement

The study was conducted with approval of the local ethics committee (reference number: 24–0014).

## Declaration of Generative AI and AI-assisted technologies in the writing process

During the preparation of this work, UA utilized ChatGPT 4 to refine language usage, correct grammar and punctuation errors to improve overall writing quality. After using ChatGPT 4, the authors (UA, BR, JW) reviewed and edited the content as needed and take full responsibility for the content of the publication.

## Funding/support

The authors declare that no financial support was received for this study.

## Declaration of competing interest

The authors declare that they have no known competing financial interests or personal relationships that could have appeared to influence the work reported in this paper.
